# *Spondias* sp: Shedding Light on Its Vast Pharmaceutical Potential

**DOI:** 10.3390/molecules28041862

**Published:** 2023-02-16

**Authors:** Érica Mendes dos Santos, Janaína Artem Ataide, Julia Cedran Coco, Ana Laura Masquetti Fava, Luiza Aparecida Luna Silvério, Ana Claudia Sueiro, Jéssica Ribeiro Alves Silva, André Moreni Lopes, Ana Cláudia Paiva-Santos, Priscila Gava Mazzola

**Affiliations:** 1 Faculty of Pharmaceutical Sciences, University of Campinas (Unicamp), Rua Cândido Portinari, 200, Campinas, São Paulo 13083-871, Brazil; 2 School of Medical Sciences, University of Campinas (Unicamp), Rua Tessália Vieira de Camargo, 126, Campinas, São Paulo 13083-887, Brazil; 3 Department of Pharmaceutical Technology, Faculty of Pharmacy of the University of Coimbra, University of Coimbra, Pólo das Ciências da Saúde, Azinhaga de Santa Comba, 3000-548 Coimbra, Portugal; 4 LAQV, REQUIMTE, Department of Pharmaceutical Technology, Faculty of Pharmacy of the University of Coimbra, University of Coimbra, Azinhaga Sta. Comba, 3000-548 Coimbra, Portugal

**Keywords:** *Spondias*, antioxidant, anti-inflammatory, antibacterial, biological activity, bioactive compounds

## Abstract

Many plants are used by the population through popular knowledge passed from generation to generation for the treatment of various diseases. However, there is not always any scientific content supporting these uses, which is very important for safety. One of these plants is the fruit of the *Spondias* genus, which during its processing generates various residues that are discarded, but which also have pharmacological properties. The focus of this review is to survey the pharmacological activities that *Spondias* genus shows, as well as which part of the plant is used, since there is a lot of richness in its by-products, such as leaf, bark, resin, seed, and peel, which are discarded and could be reused. The main activities of this genus are antioxidant, anti-inflammatory, antidiabetic, antifungal, and antiviral, among others. These properties indicate that this genus could be used in the treatment of several diseases, but there are still not many products available on the market that use this genus as an active ingredient.

## 1. Introduction

There is considerable scientific interest toward the phytotherapy potential of medicinal plants which are viewed as extremely valuable for medical and pharmaceutical applications, due to their wide implementation in traditional medicine for centuries. These plants are believed to greatly contribute to the management of several diseases, being rich natural sources of new bioactive molecules and potential alternatives to available conventional medicinal products. One example of this is the genus Spondias.

*Spondias L*. is a genus of fruit trees that belongs to the Anacardiaceae family and comprises 18 species native to tropical South America, Asia, and Madagascar [1]. Considering all of the species, we can highlight the potential for exploitation and agro-industrial and pharmaceutical uses of *S. mombin* L. (known as yellow mombin or “cajá” in Brazil), *S. dulcis* or *S. cytherea* Parkinson (ambarella, golden apple, and cajarana or cajá-manga), *S. purpurea* L. (red mombin or siriguela), *S. tuberosa* Arruda Câmara (umbu or imbu), and *S. pinnata* [2]. 

The occurrence of species with intermediate characteristics among the already-described species of *Spondias* has been reported in several studies which have tried to determine the existence of hybrids in the genus [3]. Almost all evidence of *Spondias* hybridization includes *S. mombin* as one of the putative parents, and most of them occur where one or both putative parents occur or have been introduced [4]. In Brazil, cajá–umbu or umbu-cajá (*S. mombin* x *S. tuberosa*) and umbuguela (*S. tuberosa* x *S. purpurea*) are examples of species which are considered to be natural hybrids, but which have not been taxonomically defined [5].

Members of this genus have been widely used in traditional medicine for the treatment of ailments [1], and depending on the species, different plant parts may be used. Over the years, several pharmacological activities have been attributed to *Spondias* genus; these include antibacterial, antidepressant, antifungal, antioxidant, anti-inflammatory, antiviral, antipsychotic, anxiolytic, sedative, and molluscicidal actions, among others [6,7,8,9,10,11,12,13]. These activities stem from its active compounds, such as carotenoids, caffeic acid, catechin, chlorogenic acid, ellagic acid, flavonoids, gallic acid, isoquercetin, rutin, and quercetin [6,9,14,15]. The main pharmacological applications and used plant parts of some species of genus *Spondias* will be further described in this review.

## 2. Methods

The information about the genus *Spondias* and its applications were collected from the Web of Science, Pubmed, and Virtual Library of Health databases, covering a period of 2012 to 2022. The terms used in the data search were *Spondias mombin* L. cajá, yellow mombin, tapereba, *Spondias dulcis*, *Spondias cytherea* Parkinson, cajá-manga, *Spondias purpurea* L. *S. purpurea* L. red mombin, jocote, *Spondias tuberosa* Arr. Câmara, *Spondias tuberosa*, *Spondias purpúrea*, umbu, cajá–umbu, umbuguela, *Spondias Pinnata***,** antioxidants, antibacterial agents, anti-inflammatory agents, antiviral agents, antifungal agents, antiparasitic agents, and wound healing. From this search, 95 articles were included. Some references before 2012 were included to describe concepts.

## 3. Socioeconomic Importance

Species of the *Spondias* genus are found in several regions of Brazil, but especially in the northeast, where they have considerable social and economic relevance [16]. The fruits are nutritious, highly appreciated, and commercialized *in natura* or as fruit pulp, sweets, ice creams, and juices. The trees are distinctly found in the northeast region, where the climate is semi-arid and is characterized by a long dry period, making traditional farming impossible [17]. The Umbu tree (*S. tuberosa*) is the “sacred tree of the Sertão” (typical dry biome from Brazilian northeastern region), because without it, the barren lands would be desolated [18].

In fact, northeastern Brazilian communities have low income due to the long period of drought. The trees are mainly harvested in their natural habitat or in domestic fruit farms, and have great socioeconomic importance for the semi-arid region as an alternative for food and as economic subsistence for thousands of families, generating employment and income [19]. A study conducted by Cavalcanti, et al. [20] showed that 80% of the population studied were involved in *S. tuberosa* cultivation and harvest, which shows how these species are closely linked to the income of the northeastern population [20]. According to IBGE (Brazilian Institute of Geography and Statistics), in 2020, all *S. tuberosa* production came from the northeast region and the state of Minas Gerais, and the main producer was the state of Bahia. The production represents 0.25% of all food production in Brazil (9.5 tons out of 780 tons, and 11,900 out of 1,500,000 Brazilian Reais).There are no official data on *S. mombin* production, but it is estimated that production is around 15 to 20 thousand tons of fruit per year, with production restricted to the northeast and north regions [21].

## 4. Bioactive Compounds

Bioactive compounds, in general, are mainly secondary plant metabolites [22]. Among the bioactive compounds, polyphenols are considered the most abundant, and are generally related to defense against ultraviolet radiation or aggression by pathogens or insects [23]. Several classes of polyphenols are known, and their classification and differentiation were made according to the number of phenolic rings and the structural elements that bind their molecules. Flavonoids and non-flavonoids are considered to be the two main groups among polyphenols, some of which are specific to some plant species or genera [23].

**Figure 1:**
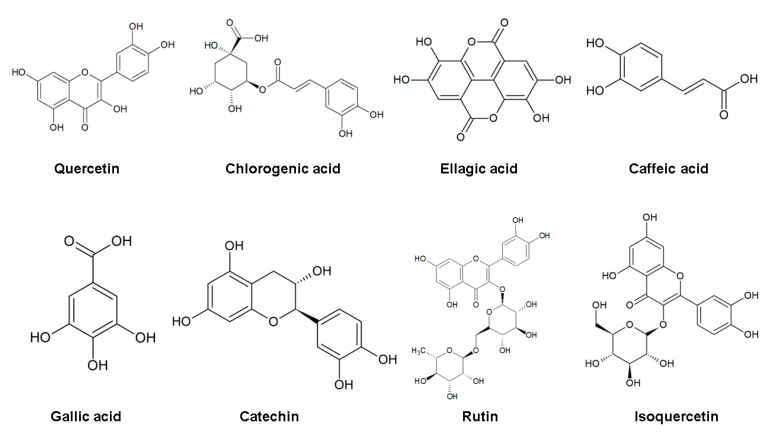
Chemical structure of the main bioactive compounds of the *Spondias* genus. Figure was created by authors using ChemSketch 1.

In this context, one of the main characteristics of the *Spondias* species is the occurrence of flavonoids, as, in addition to performing different functions in plants (including defense, ultraviolet protection and flower color), these compounds also have important bioactive properties, such as antioxidant action, that are responsible for several benefits to human health [24]. In the study by Pereira, et al. [25] the correlation between the phenolic composition of five species of *Spondias* (Anacardiaceae) was described, namely, *S. mombin*, *S. dulcis*, *S. purpurea*, *S. venulose*, and *Spondias* sp. Through this study, the authors were able to understand the evolution of the phylogenetic relationships of these species. All samples analyzed showed similar phenolic compositions, with quercetin and rutin being the main bioactive compounds. Among the phenolic constituents found in the species of *Spondias* studied, except for *S. dulcis*, quercetin 3-O-rutinoside (Figure 1) stood out. In addition, rutin (Figure 1) was considered to be a compound shared by the studied species of American *Spondias*. Other studies have shown the presence of important bioactive compounds in *Spondias* sp. such as ellagic acid [6], gallic acid, catechin, chlorogenic acid, caffeic acid [26], and isoquercetin [27], though these are in lower concentrations.

Lima, et al. [28] carried out a study to determine the presence of secondary metabolites in the crude extract of the species *Spondias* sp. and *S. tuberosa*. The obtained data showed the presence of tannins, flavonoids, steroids, and terpenoids, though the presence of alkaloids and saponins was not found. These investigations indicated promising results for the plant species studied, as the compounds found may be potentially active in biological and pharmacological applications [28].

As for antioxidant compounds, the cajá pulp showed significant amounts of phenolic compounds, in addition to high concentrations of carotenoids, tannins, and vitamin C. In this study, the authors identified five carotenoids, β-cryptoxanthin, lutein, zeinoxanthin, and α- and β-carotene, with β-cryptoxanthin being mainly responsible for the high level of provitamin A activity in the pulp. More than 37% of the recommended daily allowance of vitamin A can be supplied with just one 100 g serving of cajá pulp [29].

In recent years, many researchers have been isolating and identifying *Spondias* compounds such as epicatechin, quercetin [30], chlorogenic acid, ellagic acid [31], palmitic, linoleic, oleic, stearic, melissic, unsaturated fatty acids, and saturated fatty acids [32] for *Spondias mombin* seeds and leaves. Flavonoids, tannins [33], methylgallate, gallic acid, geraniin, sucrose quercetin, vanillicacidO-hexoside, ellagic acid, and salicylicacid, among other compounds, were found in *Spondias dulcis* leaves and stem bark [26]. Gallic acid, methyl gallate, digallic acid, and ethyl gallate were isolated from stem bark extract of *Spondias purpurea* [6] and rutin and nicotiflorin were isolated from *Spondias purpurea* pulp [7]. Palmitic acid, stearic acid, oleic acid, linoleic acid [27], gallic acid, and flavonoid [9] were isolated from *Spondias tuberosa* seeds and leaves. The leaves, flowers, and fruit of *Spondias pinnata* presented 52 essential oils such as α-Pinene, β-Myrcene, limonene, borneol, and cubenol, among others [10]. Finally, 93 volatile compounds among ethyl acetate propyl acetate, 3-Pentanol, *p*-Xylene [11], quercetin, proanthocyanidins, and ellagic acid were identified in *Spondias mombin × Spondias tuberosa* pulp [12].

Up to date, research on the *Spondias* species is scarce, and further investigation is required in order to substantiate the scientific knowledge of the bioactive compounds produced by these species, mainly in Brazilian biomes [28]. In fact, the Brazilian reality is such that although it has the greatest plant diversity in the world and many of its medicinal plants are widely known, the available information about these plants is still insufficient, especially given their potential applications, with specific emphasis on their biomedical applications [34].

## 5. Pharmacological Activity

### 5.1. S. mombin L.

*S. mombin* L. (Table 1) is a fruit tree distributed in the tropical areas of Africa, Asia, America, and in Brazil, mostly in the regions of the north and northeast [29]. The plant is known by several popular names such as cajá, cajá-mirim, and taperebá in Brazil and yellow mombin in North America [35]. The fruit has an exotic flavor and aroma, and plays an increasing role in agribusiness in the north and northeast regions of Brazil, being marketed mainly as a fresh fruit or processed pulp for use in juices, ice cream, popsicles, jellies, and yogurts [29].

Previous studies have shown that *S. mombin* L. has bioactive compounds such as chlorogenic acid [36], ellagic acid, rutin, quercetin [37], flavonoids [38], carotenoids, and vitamin C [39]. These compounds are possibly responsible for many biological activities, which include antioxidant, anti-inflammatory [40], antibacterial [37], antifungal [15], and antiviral [35] activities, among others.

*S. mombin* L. has high antioxidant activity; one study showed half-maximal inhibitory concentration (IC_50_) = 0.42 ± 0.01 mg/mL in DPPH assay (2,2-Diphenyl-1-picrylhydrazyl), and IC_50_ = 0.45 ± 0.03 mg/mL in ABTS assay (2,2’-azino-bis(3-ethylbenzothiazoline-6-sulfonic acid)) using methanolic extract from leaves [37]. Another study showed that the crude fruit juice from *S. mombin* L. obtained 93.97 ± 64.8 µmol in DPPH assay and 11.8 ± 0.2 µmol in FRAP assay (ferric-reducing antioxidant power) [38]. The high antioxidant capacity is due to the amount of phenolic compounds and flavonoid content [38].

*S. mombin* L. leaves are also used in traditional African medicine to treat neurological disorders. Studies have shown that it has sedative, anti-epileptic and antipsychotic, anxiolytic [41], and antidepressant [42] properties. These effects in the nervous system occur due to the presence of alkaloids and flavonoids in the leaves [43].

The results of this investigation show that *S. mombin* has several bioactive compounds that can contribute to pharmacological activity, which include antioxidant, anti-inflammatory, antibacterial, molluscicidal, anti-epileptic, antipsychotic, anxiolytic, and antidepressant properties, and it has low toxicity, as can be seen in Table 1. It thus presents itself as a promising alternative to be applied in the pharmaceutical and cosmetic industries.

**Table 1:** Summary of *Spondias mombin* L. activity in different parts of the plant, as described in the last few years.

Species	Activity	Part of the Plant Evaluated	References
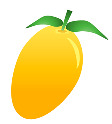 *Spondias mombin* L.	Antibacterial	Bark	[13]
		Plant	[14]
		Root, leaf, and stem bark	[15]
	Antidepressant	Leaf	[42]
	Anti-inflammatory	Leaf	[40,44]
	Antifungal	Root, leaf, and stem bark	[15]
	Antioxidant	Leaf	[37,40]
		Crude fruit juice	[38]
	Antiviral	Leaf and branches	[35]
	Anxiolytic	Leaf	[45]
	Molluscicidal	Leaf	[46]
	Sedative, anti-epileptic, and antipsychotic	Leaf	[41]

### 5.2. S. dulcis or S. cytherea Parkinson

Leaves from *Spondias dulcis* (Table 2) are used in folk medicine for skin infections and localized pain [47]. Crushed fruit is used to treat eye infections [48] and to treat itches, skin inflammation, sore throats, and ulcers [49]. Therefore, several studies aim to investigate the antimicrobial, immunomodulation, and antioxidant properties of different parts of *S. dulcis*. There are also studies which have explored the anticancer, thrombolytic, and enzyme inhibitory effects of *S. dulcis*.

Islam, Ahmed, Manik, Wahid and Kamal [49] performed antimicrobial screening of *S. dulcis* fruit and leaves via the disc diffusion method. Methanolic extracts of fruit and leaves were produced and then partitioned with dichloromethane and chloroform. All six *S. dulcis* extracts demonstrated moderate antimicrobial activity against Gram-positive and Gram-negative bacteria, such as *P. aeruginosa*, *E. coli*, *S. aureus*, *Shigella boydii*, and *Salmonella paratyphi*, and weak activity against *Candida albicans*. The same authors investigated the thrombolytic activity of *S. dulcis* extracts. All six extracts were significantly more thrombolytic than the negative control; however, the highest clot lysis induced by *S. dulcis* (25%) was lower than the positive control (50%).

*S. dulcis* presents other enzyme inhibitory effects, such as anti-α-glucosidase and anti-α-amylase, responsible for the digestion of carbohydrates in the intestine. The inhibition of these enzymes is one of the mechanisms that reduces postprandial hyperglycemia in diabetic patients [50]. Ethanolic extracts from *S. dulcis* leaves [51] and fruit [52] presented anti-α-glucosidase activity with IC_50_ of 45.52 µg/mL and 4.73 µg/mL, respectively.

*S. dulcis* fruit aqueous extract presented anticancer activity against melanoma, a highly aggressive type of skin cancer [53]. The extract inhibited the in vitro proliferation, migration, and invasion of melanoma cells. In vivo, the tumor significantly reduced when treated with intraperitoneal injections of *S. dulcis* extract at 450 mg/kg for 15 days. *S. dulcis* reduced cyclooxygenase (COX-2) enzyme expression and downregulated CD133 glycoprotein, both associated with cancer growth, invasion, and metastasis. Further studies are required to identify the compounds responsible for the observed anticancer activity and to develop novel chemotherapeutic treatments for melanoma.

Ethanolic extracts of *S. dulcis* leaves and fruits presented IC_50_ for DPPH scavenging assay of 14.22 µg/mL and 27.14 µg/mL, respectively [51,52]. Moreover, methanolic extracts were more effective antioxidants, with an IC_50_ of 5.37 ug/mL for the leaves and IC_50_ of 1.91 µg/mL for the fruit [49]. Methanol was also among the best solvents to extract phenolics from *S. dulcis* fruit (659.74 mg GAE/g) (gallic acid equivalent), leaves (609.71 mg GAE/g), and stem bark (657.31 mg GAE/g), and a positive correlation between antioxidant activity and total phenolic contents was observed [26].

In summary, *S. dulcis* extract and isolated fractions present relevant in vitro and in vivo pharmacological activity, with anticancer, antioxidant, antimicrobial, antimutagenic, antigenotoxic, thrombolytic, immunomodulation, and enzymatic inhibitory properties. Therefore, *S. dulcis* may be studied as a novel or synergistic approach to treat infections, skin disorders, and high-incident diseases, such as Alzheimer’s, cancer, diabetes, and obesity. The authors have already demonstrated the promising practical applications of this plant in food technology and nanoscience for environment care. However, further studies are needed to apply the pharmacological potential that this species may offer.

**Table 2:** Summary of *Spondias dulcis* activity in different parts of the plant, as described in the last few years.

Species	Activity	Part of the Plant Evaluated	References
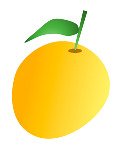 *Spondias dulcis* or *Spondias cytherea* Parkinson	Anticancer	Fruit	[53]
	Antimicrobial	Fruit	[49]
		Leaf	[49,54]
		Peel	[55]
	Antimutagenic and antigenotoxic	Stem bark	[56]
	Antioxidant	Fruit	[49,52]
		Leaf	[26,49,51,57]
		Stem bark	[26,56]
	Enzymatic inhibition	Fruit	[52]
		Leaf	[26,51]
		Pomace	[58]
		Stem bark	[26]
	Immunomodulation	Pulp	[59]
	Thrombolytic	Fruit	[49]
		Leaf	[49]

### 5.3. S. purpurea L.

*S. purpurea* L. (red mombin, Mexican plum, ciriguela) (Table 3) [60] is a tropical medium-sized tree, whose fruit is an ellipsoid drupe which evidences a yellowish pulp and a thin yellow or reddish peel [61]. The fruit, which is consumed in both fresh and processed states, as juices, fermented beverages, wines, ice creams, and jams [60], constitutes a source of phenolic compounds, including tannins, phenolic acids, and flavonoids [61,62]. This plant represents a valuable source of income for many rural properties, due to its recognized sensorial and nutritional values, as well as the low cost of production [63]. In this context, the fruits and fractions of this species have been used in popular knowledge for centuries towards the management of a broad array of diseases, including diabetes, inflammatory reactions, diarrhea, and gastritis. These applications are largely due to its antioxidant, anti-inflammatory, and photoprotective activities [62], which have created interest in this species to be employed as promising ingredients of medical and cosmetic formulations.

Just as with the other genus *Spondias* species, *S. purpurea* L. evidences recognized antioxidant activity, which is mostly attributed to the presence of bioactive flavonoids and also to the phenolic content of the corresponding extracts [6,7,64]. Such evidence has been supported by several studies. Examples include the methanolic extract of *S. purpurea* L. crude peel [62], the hexane-based extract of the leaves of *S. purpurea* L. [65], and the hexane extract of the fruit of *S. purpurea* L. [66]. It must be highlighted that there are differences in the antioxidant properties and in the composition of functional active agents among all of the ecotypes [63,64].

In another context, the hexane and the ethanolic extracts of the leaves of *S. purpurea* showed the ability to reduce the area of ulcerative lesions in an in vivo ethanol-induced gastric ulcer model. These results were compared with the nonsteroidal anti-inflammatory control drug (indomethacin), which revealed inferior percentages of protection. Thereby, hexane and the ethanolic extracts of the leaves of *S. purpurea* impacted on the minimization of ulcers, the enhancement of the levels of reduced glutathione, and the lowering of the tumor necrosis factor, clearly emphasizing the anti-ulcerogenic properties of this plant, indicating it as a potent anti-ulcer agent [65].

Curiously, recent studies have also been devoted to several other relevant applications of this species, looking at its inherent potential applications and greatly increasing the interest in this enriched natural source of bioactives. An example is the capacity of *S. purpurea* L. seed flour as a highly innovative ingredient that can substitute common refined (white) wheat flour in cakes due to its technological properties. What is more, the use of *S. purpurea* L. seed flour provides longer preservation of the product because of its antioxidant attributes, and also enhances the nutritional content of the food product [60].

Overall, studies with *S. purpurea* are scarce and constrained to fruits and gum exudates [65]. A variety of ecotypes that have not been characterized exist, and their adequate selection process, pertaining to their valuable functional properties, will enable the constitution of advanced protocols for this plant and fruit [63,64]. More studies should be performed to thoroughly investigate this plant and to thereby select or produce *S. purpurea* L. ecotypes with a higher content of antioxidant bioactives and more beneficial health properties [64], aiming for the significant implementation of the several inherent medical and cosmetic properties that this plant may offer.

**Table 3:** Summary of *Spondias purpurea* L. activity in different parts of the plant, as described in the last few years.

Species	Activity	Part of the Plant Evaluated	References
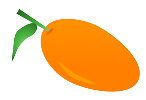 *Spondias purpurea* L.	Antidiabetic	Seed	[67]
	Antihyperlipidemic	Seed	[67]
	Antimicrobial	Leaf	[68]
	Antioxidant	Fruit	[69]
		Leaf	[65]
		Peel	[62,63]
		Pomace	[70]
		Pulp	[63,71,72]
	Anti-ulcer	Leaf	[61,73]
	Photoprotective	Peel	[65]

### 5.4. S. tuberosa Arr. Câmara

*S. tuberosa* Arr. Câmara (umbu) (Table 4) is an endemic fruit found in the caatinga, an exclusive Brazilian biome, characterized by semi-arid weather. The fruit belongs to the *Anacardiaceae* family, and it is characterized by its oval or ovoid shape. Furthermore, the pulp contains juice, with a sweet taste, which is full of antioxidant bioactives (phenolic compounds and carotenoids) and vitamin C [7,27,74,75]. Thus, there is interest in the manufactured by-products of this fruit; in fact, frozen pulps, nectar, and liqueurs are the most produced products in this industry [76].

Besides the manufacturing interest, its antioxidant activity has been targeted pharmacologically for cosmetic and medical use. Antioxidant compounds in the *Spondias* species are particularly relevant for the prevention and improvement of diseases related to oxidative stress. In the case of *S. tuberosa*, the presence of tannins and phenols in its peel has been reported [77,78], as well as organic acids in the peel, seeds, and pulp [78]. As well as this, total phenolic content in umbu was found to be high in the seed, peel, and pulp, consecutively.

In addition, umbu methanolic extract also demonstrated higher antioxidant activity when compared to ethanol and acetone extracts in studies where the three different extracts revealed antioxidant efficacy [79]. Additional studies evidenced lower phenolic compound detection using the liquid extract, suggesting that the components present in umbu are polar. In this study, the antioxidant activity of the liquid extract was not reduced, and, as a matter of fact, it was curiously higher [8]. Overall, the previous studies confirm the possible existence of polar bioactives that may increase the antioxidant capacity, but which may not necessarily be phenolic compounds.

In vivo studies conducted with diabetic rats treated with hydroalcoholic extract of umbu demonstrated an enzymatic antioxidant defense, which led to the conclusion that the plant may act as a radical capable of reducing oxidative stress in diabetic rats, protecting their functions, especially of the liver, from hepatic tissue damage [80].

In summary, many studies have proven the antioxidant capacity of *S. tuberosa* through DPPH, ABTS, ORAC, ascorbic acid, beta-carotene bleaching assay, and other tests [69,81,82]. According to these data, it is possible to confirm that the antioxidant activity from *S. tuberosa* is related to the phenolic content, even though this is lower than other fruits and plants. Moreover, the fruit part used in the studies may present different results of antioxidant activity intensity, in the same way that the form of liquid extract interferes in this activity. Such a result is attributed to the distribution of the phenolic content throughout different parts of the plant.

Umbu ethanolic seed extract presents inhibitory activity in acetylcholinesterase (AChE) [77]. This activity supports the use of umbu for degenerative conditions, such as Alzheimer’s, given the antioxidant properties associated with AChE inhibition, which promote protection against free radicals [83].

The hexane extract of umbu leaves demonstrated the capability of inhibiting the growth of *C. albicans* and *C. glabrata*, contrary to *C. parapsilosis* and *C. krusei*, in which no fungicidal activity was detected. The extract was able to cause mitochondrial alteration and lysosomal compromise in *C. glabrata* functions. In this case, the hexane influenced bioactive absorption by the selection of a specific hyperoxide, 3-9-galactoside of quercetin, even though some antioxidant compounds must have been extracted due to the antioxidant capacity tests which characterized the solution as an antioxidant medium. Even considering these effects, the extract was classified by the authors as non-hemolytic [9].

According to in vitro tests in agar, *S. tuberosa* extract promoted a significant diffusion with Gram-negative bacteria, such as Klebsiella pneumonia, Serratia marcescens, Pseudomonas aeruginosa, Proteus mirabilis, Morganella morganii, Serratia liquefaciens, and Enterobacter cloacae. In comparison to ciprofloxacin, the minimum inhibitory concentration was (MIC 7.8 UG/ML) and a marked difference in antibacterial activity was found for the extract, which showed higher MIC values [37].

Bioactives in *S. tuberosa* branch extract, as mentioned before, contain significant antioxidant activity, demonstrating gastroprotective effects. In an in vitro gastric ulcer model, the use of branch extract of *S. tuberosa* improved the lesion when compared to the control group (lansoprazole). This is attributed to the ability of the antioxidant to capture the free radicals, and reduce the lesion in the gastric mucosa [84].

Due to the aforementioned discussion, *S. tuberosa* presented a promising source of bioactive compounds, endowed with relevant antioxidant, antidiabetic, anti-acetylcholinesterase, antibacterial, antifungal, gastroprotective, and non-cytotoxic documented properties. With this in mind, the umbu fruit presented a promising alternative for conventional therapies.

**Table 4:** Summary of *Spondias tuberosa* activity in different parts of the plant, as described in the last few years.

Species	Activity	Part of the Plant Evaluated	References
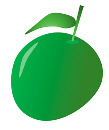 *Spondias tuberosa*	Antidiabetic	Pulp	[85]
		Peel, pulp, and seeds	[12]
	Antioxidant	Pulp	[72,78,85]
		Peel, pulp, and seeds	[12]

### 5.5. S. pinnata

*S. pinnata* are decidua trees, also known as “kedondong hutan”, and can reach 10 to 25 m with full growth. These trees are commonly found in primary and secondary forests [1,2,86]. They are characterized by a smooth bark, with irregular cracks and a gray to reddish-brown color, and the sap is transparent and viscous. Table 5 evidences some significant works related to the diverse kind of approaches using this promising plant, ranging from the most commonly used part in research—the bark—to other parts of the tree that have also been explored, such as resin, leaves, and fruit, among others. *S. pinnata* is distinguished from the other members of the genus, mainly by its short and pedicellate flowers, and by its endocarp characterized by a dense, fibrous, and hard outer layer. The roots, leaves, and fruit, among other parts of this tree, show numerous beneficial activities and therefore have been widely used in medicine and pharmacy in many countries [86]. The main pharmacological activities found include antioxidant, antimicrobial, anticancer, hypoglycemic, and anti-inflammatory activities, and are discussed below.

Hazra, et al. [87] evaluated the in vitro antioxidant properties of stem bark extract from *S. pinnata*. The antioxidant activities were evaluated for the scavenging of superoxide anions, hydroxyl radicals, hydrogen peroxide, nitric oxide, peroxynitrite, hypochlorous acid, and singlet oxygen, as well as for iron chelating capacity. The evaluated IC_50_ values are 13.46, 112.18, 44.74, 24.48, 716.32, 127.99, and 58.07 μg/mL, respectively. These data are relevant as some illnesses are related to oxidative stress produced by free radicals. Thus, if the free radicals can be scavenged by antioxidants, many illnesses and ailments in living systems can be avoided, such as ageing, cardiovascular and inflammatory diseases, atherosclerosis, and cancer, among others.

In another work, Jain [2] verified the antioxidant and antibacterial properties of extracts from *S. pinnata* leaves, by using ethyl acetate and ethanol extracts which exhibited high scavenging activities. The highest flavonoid amount was obtained by ethyl acetate extract, while ethanol extract presented the highest total phenolic amount: 86.53 mg of quercetin equivalent (QE)/g extract and 27.76 mg GAE/g extract, respectively. Regarding the antibacterial activity, all of the extracts evaluated exhibited a variation from 8.33 to 28.67 mm of the inhibition zone. The ethanol extract tested against *Staphylococcus aureus* showed the lowest minimum bactericidal concentration (MBC) and MIC values of 3.5 and 2.0 mg/mL, respectively. Thereby, all of the extracts of *S. pinnata* leaf evaluated can be considered to have relevant formulations with antibacterial and antioxidant properties for pharmaceutical and medical applications.

The anti-inflammatory, antimicrobial, and cytotoxic effects of the fruit peel essential oil from *S. pinnata* were studied by Li, et al. [88]. These bioactivities demonstrated anti-inflammatory activity by preventing nitric oxide production by endotoxin-introduced RAW 264.7 cells at concentrations of 0.08%, without any effect on cell viability. These bioactivities have also shown relative antibacterial activity against five pathogenic strains, (being *Staphylococcus aureus*, *Acinetobacter baumannii*, *Pseudomonas aeruginosa*, *Aspergillus fumigatus*, and *Candida albicans*), with a minimal fungicidal concentration (MFC) and MIC of 32 and 16 µg/mL, respectively. These values were 32-fold higher than the inhibition effect attained with *Aspergillus fumigatus* and tigecycline (a positive control), with a MBC and MIC of 1024 and 512 µg/mL, respectively. In addition, there was cytotoxic activity against five tumor cell lines (i.e., HL-60, SMMC-7721, A-549, MCF-7, and SW480 cell lines).

*S. pinnata* is also attracting growing attention regarding its application as a therapeutic anticancer source. Ghate, et al. [89] investigated the activity of bark extracts derived from this plant in inducing apoptosis in human breast adenocarcinoma and human lung adenocarcinoma cell lines (MCF-7 and A549, respectively). Results evidenced the anticancer potential of *S. pinnata* against cancer cells by inducing apoptosis through the modulation of Bcl-2 family proteins. Specifically, this extract exhibited cytotoxicity to both MCF-7 and A549 and cell lines (IC_50_ of 149.34 and 147.84 μg/mL, respectively), while, for normal cells (i.e., human lung fibroblast WI-38 cells), no cytotoxicity was observed (IC_50_ of 932.38 μg/mL).

With the aforementioned discussion in mind, *S. pinnata* constitutes a promising source of bioactive compounds, endowed with relevant and documented antioxidant, thrombolytic, antimicrobial, antidiabetic, and anticancer properties, being, thereby, presented as a promising alternative for conventional therapies.

**Table 5:** Summary of *Spondias pinnata* activity in different parts of the plant, as described in the last few years.

Species	Activity	Part of the Plant Evaluated	References
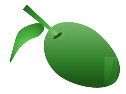 *Spondias pinnata*	Anti-inflammatory	Bark	[90]
	Antioxidant	Bark	[91]
	Antimicrobial	Resin	[92]
	Cellular immunity and phagocytosis	Leaf	[93]
	Hepatoprotective	Bark	[91]

### 5.6. S. mombin × S. tuberosa

*S. mombin* × *S. tuberosa* (cajá–umbu) (Table 6) is a fruit from the northeast of Brazil and is the result of the natural crossing between the species of *S. mombin* (cajá) and *S. tuberosa* (umbu). In the same way as umbu, the cajá–umbu fruit belongs to the *Anacardiaceae* family and is characterized by its oval shape, thin peel, and yellow color. Furthermore, the fruit has a sweet taste, which attracts the food industry for fruit extraction and the production of juices, candies, and ice cream [27].

Besides its attractive taste, cajá–umbu contains important bioactives such as vitamin C, an important antioxidant, and other carbohydrates [27]. In fact, antioxidant activity was the prevalent activity studied in the literature for cajá–umbu. Phenolic compounds were found in the pulps of cajá–umbu at a total concentration of 62.08 ± 0.069. According to the DPPH test, cajá–umbu showed higher antioxidant activity when compared to the commercial antioxidant butylhydroxytoluene [78]. Accordingly, other authors obtained a DPPH result of 10.53 ± 0.19 mmol Trolox/g sample and FRAP of 0.31 d 0.01 mmol Trolox eq/g sample. These results were considered to be statistically significant, confirming the antioxidant activity [12].

More recently, the ORAC test (oxygen radical absorbance capacity) proved that residues of cajá–umbu, in an aqueous extract, were highly effective in their oxygen radical absorbance capacity. However, after oral consumption and gastrointestinal digestion, the antioxidant activity was reduced due to phenolic compound destabilization in intestinal pH. Even with the possible destabilization of the molecule by intestinal pH, the antioxidant capacity may not completely disappear, since the fermentation in the intestinal environment by bacteria may bio-convert the phenolic compounds [85].

Furthermore, cajá–umbu also demonstrated moderate to low inhibition of alpha-glucosidase, as with many other fruits such as cashew apple, canafistula, cupuassu, soursop, manguba, strawberry [85], acerola, and pitanga [12], due to the flavonoids and phenolic compounds present. Interestingly, the concentration of flavonoids in cajá–umbu, which presents an important role in alpha-glucosidase inhibition, is present in lower values compared to the other fruits. In fact, only quercetin was significantly detected (3.38 ± 0.01 mg aglycone/100 g), and it showed no correlation with the activity of alpha-amylase inhibition. Besides these results, the intensity of enzymatic inhibition by cajá–umbu makes it a promising fruit for antidiabetic use [12].

In summary, cajá–umbu is a promising source of antioxidant activity, which could be relevant to treat diabetes, for instance. With this in mind, the cajá–umbu fruit is a promising alternative for conventional therapies, and holds valuable information that should promote other experimental studies.

**Table 6:** Summary of *Spondias Spondia tuberosa* Arruda x *Spondia mombin* activity in different parts of the plant, as described in the last few years.

Species	Activity	Part of the Plant Evaluated	References
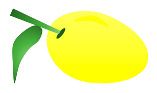 *Spondia tuberosa* Arruda × *Spondia mombin*	Anti-acetylcholinesterase	Pulp	[83]
	Antibacterial	Leaf	[37]
	Antidiabetic	Pulp	[81]
		Bark	[80]
	Antifungal	Leaf	[9]
	Antioxidant	Pulp	[71,78,79]
	Cytotoxic	Bark	[94]
		Seed	[95]
		Peel, pulp, and seeds	[77]
	Gastroprotective	Leaf	[84]

## 6. Conclusions

The *Spondias* genus has long been used in traditional medicine for various ailments. Different parts of this plant have been used in treatments, such as leaves, bark, fruits, branches, and roots. In this genus, there are different classes of bioactive compounds, including phenolics, flavonoids, carotenoids, quercetin, ellagic acid, and chlorogenic acid, among others, which are responsible for its biological activities. Among these activities, the ones that stand out are antioxidant, anti-inflammatory, anti-ulcer, antihyperlipidemic, hepatoprotective, thrombolytic, antimicrobial, antidiabetic, and anticancer. *S. mombin* L. has high antioxidant activity, it is used to treat neurological disorders in traditional African medicine, and in recent years it has begun to be further studied. *S. dulcis* is one of the most studied species among the *Spondias* sp. and its research is in regard to skin diseases, such as infections, and others illnesses, such as Alzheimer’s, cancer, and diabetes. Studies with *S. purpurea* are rare, although it has been used for centuries in popular knowledge for diseases such as diabetes, diarrhea, and gastritis. *S. tuberosa* and *S. pinnata* are two of the least studied species, but show promising gastroprotective, antifungal, antibacterial, and antioxidant properties, among others. The most common activity reported in the literature for *S. mombin* × *S. tuberosa* is antioxidant, and it is a viable replacement for commonly utilized treatments. However, in regard to the limitations of this study, articles addressing the use of residues of this genus are lacking in the literature, especially for *S. tuberosa* and *S. pinnata.* As well as this, there are not many products on the market nor articles in the literature that actually use this genus as a final product. Therefore, the development of tests with other types of by-products of this genus and the production of pharmaceutical and cosmetic products are future perspectives of advances with the *Spondias* genus.

## Data Availability

No new data were created or analyzed in this study. Data sharing is not applicable to this article.
